# Reinforcing effects of aminosilane-functionalized h-BN on the physicochemical and mechanical behaviors of epoxy nanocomposites

**DOI:** 10.1038/s41598-020-67759-z

**Published:** 2020-06-30

**Authors:** A. S. Mostovoy, M. A. Vikulova, M. I. Lopukhova

**Affiliations:** Yuri Gagarin State Technical University of Saratov, Polytechnichskaya St., 77, Saratov, Russia 410054

**Keywords:** Chemical engineering, Nanoparticles, Mechanical properties, Nanocomposites, Polymer chemistry, Polymer characterization

## Abstract

The results of this study confirm the possibility of the directional regulation of operational properties of epoxy composites by the use of small additives of hexagonal boron nitride (h-BN), providing the creation of epoxy composites with high performance properties. The effectiveness of h-BN surface modification by γ-aminopropyltriethoxysilane and the formation of strong chemical bonds at the polymer matrix/filler interface has been proved, which ensures an increase in physico-mechanical characteristics of epoxy composites: bending stress increases by 142% and bending modulus increases by 52%, strength increases by 53% and tensile elastic modulus increases by 37%, toughness increases by 400% and Brinell hardness increases by 96%, compared with an unfilled plasticized epoxy composite.

## Introduction

Research in the field of polymer modification with functional additives is widely used to create polymer materials with a given set of consumer characteristics. So, to give polymer composite materials and products made from them enhanced functional characteristics, various modifying additives are used, for example, graphite, CNTs, TEG, graphene or metal oxides^1–14^. The use of nanocomposites is determined by their unique properties, which are due to the huge specific surface and high surface energy of nanoparticles. Nanometer particles, unlike micro- and larger inclusions, are not stress concentrators, which contributes to a significant increase in the mechanical properties of nanocomposites^1–3,7^.

However, the addition of nanomaterials into epoxy compositions only slightly increases the strength of composites and in some cases even decreases it. This is due to small adhesion between nanomaterials and the polymer matrix and to the fact that it is often energetically more profitable for nanomaterials to agglomerate with each other. As a result, to improve adhesion interaction with the polymer matrix and to improve dispersion (reduce agglomeration) hardening nanomaterials are modified in various ways: by plasma treatment, by acid treatment, amines, etc.^6–9^.

A promising method of surface modification of nanomaterials is direct fluorination, i.e. the effect of fluorine gas mixtures at temperatures from room temperature to 400–500 °C. Fluorination also leads to improved solubility of nanomaterials in various solvents. The effect of CNTs modified by direct fluorination on the properties of epoxy composites was shown in^7^. The addition of 0.1 mass % fluorinated CNTs improves physical and mechanical properties: 50% increase in strength and 74% increase in modulus of elasticity during tension, 60% increase in strength and 66% increase in modulus of elasticity during bending, and besides, abrasive wear decreases by 33%.

In this research^6^, aminopropyltrimethoxysilane was incorporated as an interfacial modifier on the surface of graphene (Gr) nanoplatelets. The addition of functionalized Gr enhanced the tensile strength and strain to failure only at low contents (i.e., 0.05 wt%). Besides, this decreased the coefficient of friction and wear rate by approximately 40% and 68%, respectively. Enhanced tensile, compressive, and wear properties in the functionalized Gr–epoxy samples were observed compared to those in the Gr–epoxy samples.

The authors^15,16^ proposed a technology for combining an epoxy oligomer with a nanoscale filler, which consists in joint grinding using microwave-assisted ball milling. Such modification ensures the chemical interaction of the epoxy oligomer with a titanium hydride, proved by IR spectroscopy, which could not be obtained by conventional grinding using a ball mill. The proposed technology leads to improved compatibility of TiH_2_ nanoparticles with the epoxy oligomer, their uniform distribution in the composition, which provides an increase in the physicomechanical characteristics of polymer composite materials.

In^17^, zinc borate nanoparticles coated with a silicon oxide were obtained to create fire-resistant composites. It was found out that zinc borate nanoparticles modified with a silicon oxide are more uniformly distributed in the epoxy resin compared to the unmodified additive.

The incorporation of nanomodifiers into the epoxy composition leads to a change in its structure to a greater or lesser extent compared to the unfilled cured epoxy binder. Besides, the matrix affects the nature of the distribution of nanoparticles by volume, which is especially important in cases when it comes to the separation of the nanomodifier. The matrix determines the size and the shape of the resulting nanoparticles. Their interaction with epoxy resin forms interphase layers. Undoubtedly, all these factors affect the properties of epoxy nanocomposites^1–11,15–17^.

The various nanosystems will produce different effect in the same polymer matrix due to the diversity in reactivity, compatibility, and dispersion stability.

Currently, there is still a lack of understanding on the suitability of functionalization of h-BN in the epoxy matrix with different composition. Issues related to the influence of aminofunctionalized h-BN on the structure formation processes, on the structure and operational characteristics of polymer composite materials are still not completely studied, which predetermines the goal of the study of the given work.

The aim of this work is to increase physicochemical and mechanical properties of epoxy composites using nanodispersed particles of hexagonal boron nitride functionalized with aminosilane.

## Materials and research methods

The compositions were developed on the basis of epoxy resin ED*-*20 (GOST 10587-93), because it has low viscosity, a narrow limit of the epoxy groups content, stability of physico-chemical properties, epoxy equivalent—195–216 g/mol, epoxy groups content 20.0–22.5%, manufactured by CHIMEX Limited (Russia). As a hardener of an epoxy oligomer, an amine type hardener was used—polyethylene polyamine (PEPA) (TS 6-02-594-85) manufactured by CHIMEX Limited (Russia), capable of forming a three-dimensional network structure without heating, molecular mass—230–250 g/mol, amine number—1,250 mg KOH/g^18,19^.

The tri-2-chloroethyl ether of orthophosphoric acid (TCEP) with purity of 95–99% which is an orthophosphoric acid and ethylene chlorohydrin ester, manufactured by Xuancheng City Trooyawn Refined Chemical Industry Co., Ltd (China) was used to plasticize epoxy composites.

The choice of TCEP is due to the presence of combustion inhibitors—phosphorus (10.8%) and chlorine (36.9%). During thermal decomposition of the composite, the presence of phosphorus provides an increase in the yield of carbonized structures, which are a physical barrier for the inter-diffusion of the oxidant and combustible gases to the combustion zone. Chlorine, being formed during the pyrolysis of the compositions, gets into the gas phase and dilutes the combustible gases, reducing the concentration limit of ignition, which, in general, reduces the flammability of epoxy composite^18^.

Hexagonal boron nitride (h-BN) manufactured by Nanoamor, Inc. (USA) which resembles graphite in its structure was used as a nanostructural additive. The main difference between the crystal structure of h-BN and graphite is the position of planes with strong interatomic bonds relative to each other. If in graphite every second atomic layer is shifted relative to other layers by a constant value, then in h-BN the hexagons formed by three boron atoms and three nitrogen atoms are directly under each other. Moreover, each boron atom in one atomic layer has a nitrogen atom in the other layer as its nearest neighbor^20^.

To impart hydrophilicity to the surface of h-BN and to make possible its sizing, h-BN was annealed at 1,100 °C for 1 h. The surface of h-BN was functionalized with a γ-aminopropyltriethoxysilane (APTES) coupling agent, manufactured by the Penta-91 company (Russia). For this purpose, 0.5 g of h-BN was dispersed in 100 ml of the H_2_O—APTES (95–5) solution with ultrasonic homogenizer for 10 min. The suspension was kept under reflux at 80 °C under constant low-speed stirring at 100 rpm for 12 h. The pH of the mixture was adjusted to 5. We selected an acidic media (CH_3_COOH) in order to increase the level of silanol formation and to decrease self-condensation reactions between the hydrolyzed silanol groups. To remove the remaining silane compound around the h-BN particles the resulting suspension was centrifuged and washed twice with H_2_O. Then, the product was dried (80 °C for 5 h) in the laboratory oven.

The ratio of epoxy oligomer, plasticizer and hardener was previously determined experimentally: 100 parts by mass of ED-20, 40 parts by mass of TCEP and 15 parts by mass of PEPA^21,22^. In plasticized epoxy composition h-BN was added as a modifying agent (0.01–1.0 parts by weight). To increase the uniformity of distribution and hinder the aggregation of h-BN particles, as well as the activation of its surface and binder, ultrasonic treatment of the composition was used. The parameters of the ultrasound exposure: frequency—22 ± 2 kHz, power—400 W, duration—60 min^23^. The mixture was degassed at 25 ± 5 °C for 30 min under vacuum before curing.

The preparation method of the composition: addition of TCEP to epoxy oligomer → mechanical stirring of the composition (ED-20 + TCEP) for 15 min → addition of h-BN to the epoxy composition (ED-20 + TCEP) → mechanical stirring of the composition for 15 min → further dispersion by sonication for 1 h (400 W; 22 kHz) → addition of the curing agent PEPA to the mixture at 25 ± 5 °C → degasification to remove air bubbles at 25 ± 5 °C for 30 min under vacuum before curing → pouring into a mould and curing at 25 ± 5 °C for 24 h → heat treatment of the epoxy composite at 100 ± 5 °C for 2 h; 120 ± 5 °C for 2 h^19^.

The research was carried out using the following methods: determination of bending stress and flexural modulus [ISO 178: 2010]; determination of strength and modulus of tensile elasticity [ISO 527-2: 2012]; determination of compressive strength [ISO 604: 2002]; determination of impact strength [ISO 179-1: 2010]; determination of Brinell hardness [ISO 2039-1: 2001]; determination of heat resistance according to Vicat [ISO 306: 2004]; change in mass, rate of change in mass and magnitude of thermal effects during the heating of the samples was studied using the method of thermogravimetric analysis with the help of a derivator of the “Paulik—Paulik—Erdei” system of the MOM brand Q-1500D under the experimental conditions: weight—100 mg, medium—air, heating interval—up to 800 °C, heating rate—10 °C/min, relative error does not exceed 1%; the study of the surface morphology of the samples was carried out using a Tescan VEGA 3 SBH scanning electron microscope; determination of thermal conductivity and thermal resistance was carried out using the ITP-MG4 "100" instrument [ISO 22007-2: 2015]; FT-IR spectroscopy of TEG particles was carried out using the Shimadzu IRTracer-100; X-ray phase analysis was performed using ARL X’TRA X-ray diffractometer^18^; determination of the curing kinetics of the epoxy composition was carried out according to the method described in^24^; the specific surface area of the samples was determined by means of the Quantachrome Nova 2200 specific surface area and porosity analyzer using the initial portion of the isotherm of physical nitrogen sorption (99.999%). The studied samples were placed in a cell preliminarily calibrated with respect to internal volume at a temperature of liquid nitrogen (about 78 K), which was degassed in vacuum to a constant mass at a given temperature (150 °C) for 3 h. After that, the cell was installed in the device and the isotherm of nitrogen adsorption was measured in the pressure range 0.03–0.3 P/Po. The specific surface of the test sample was calculated by the BET method with the Quantachrome Nova 2200 software.

## Experimental results and discussion

**Figure 1:**
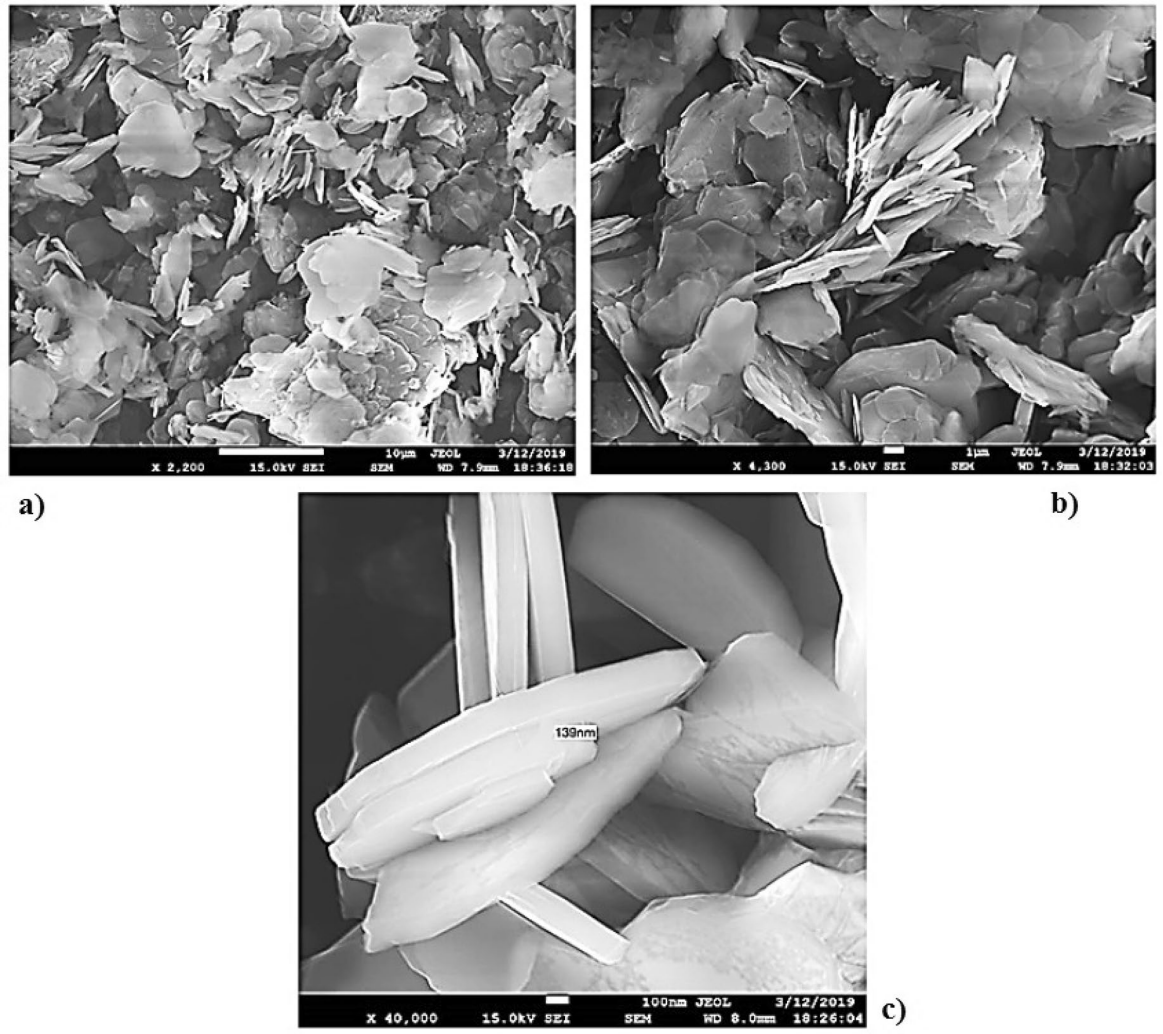
SEM of h-BN particles: (**a**)— × 2,200; (**b**)— × 4,300; (**c**)— × 40,000.

The surface morphology of the dispersed h-BN powder was analyzed by scanning electron microscopy, Fig. 1. h-BN particles are large aggregates consisting of smaller particles of scaly shape with irregular-shaped edges, about 140 nm thick.

**Figure 2:**
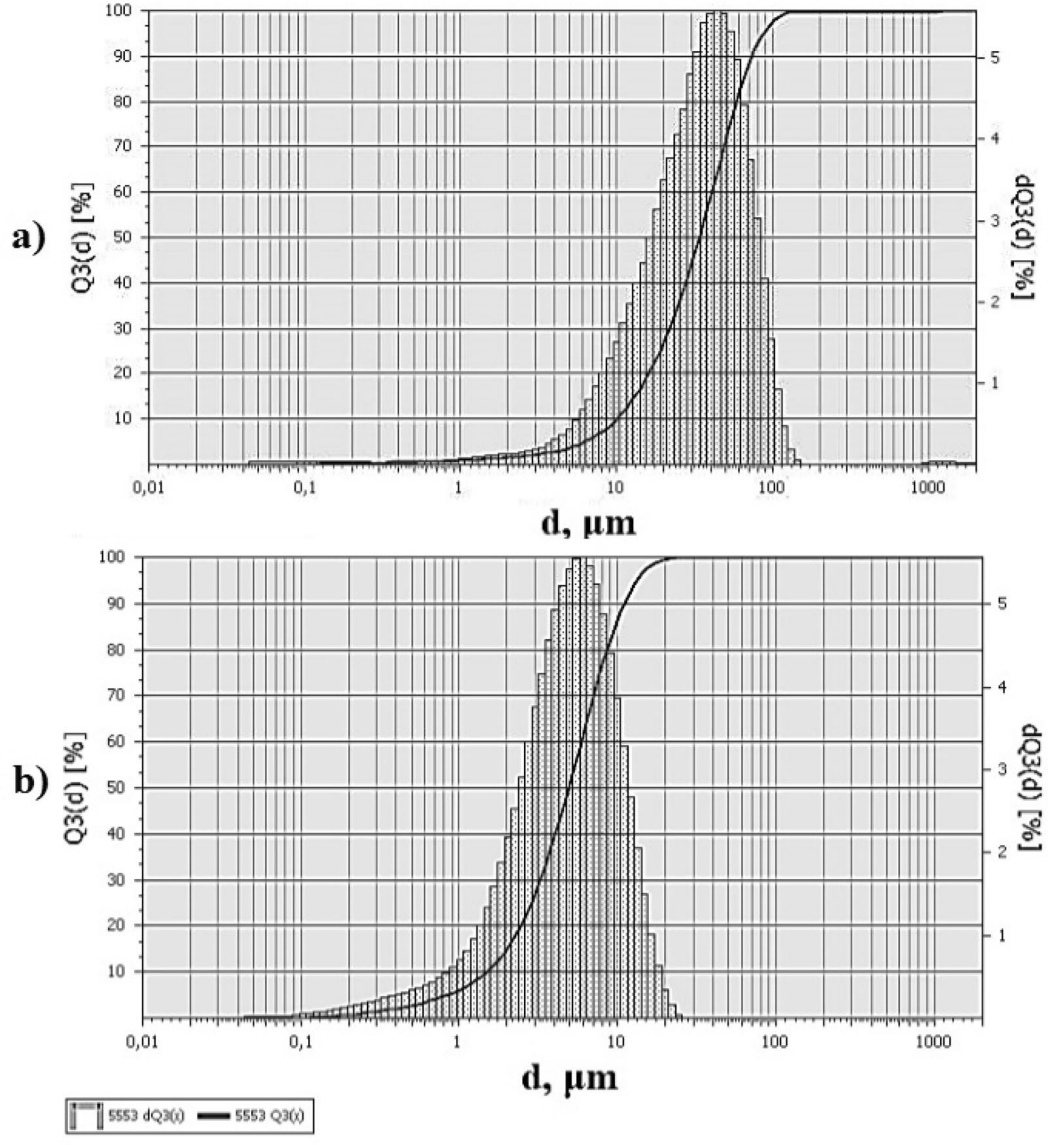
Fractional composition of h-BN particles: (**a**)—pristine h-BN; (**b**)—h-BN after APTES treatment.

The fractional composition of pristine h-BN is characterized by the unimodal distribution of particles and is represented by particles from 1 to 100 μm, with a predominant number of particles with sizes of 40 μm, Fig. 2a.

The specific surface area of h-BN particles, determined by using the specific surface area and porosity analyzer (Quantachrome Nova 2200) and the low-temperature nitrogen adsorption method, is 20.3 m^2^/g.

As a polymer matrix we used a previously developed composition^21,22^, consisting of 100 parts by weight of epoxy resin brand ED-20, 40 parts by weight of TCEP and 15 parts by weight of a PEPA hardener.

TCEP performs the functions of both a plasticizer and a flame retardant. In this case, bending stress increases by 32% and toughness is 2.6 times higher, the flammability index–oxygen index (CI) increases from 19 to 27% by volume, which ensures the transition of the material to the class of hardly flammable^21,22^. In addition, the presence of a chemical interaction of TCEP with an epoxy oligomer during curing was previously proved^22^.

In plasticized epoxy composition h-BN was added as a modifying agent (0.01–1.0 parts by weight).

**Figure 3:**
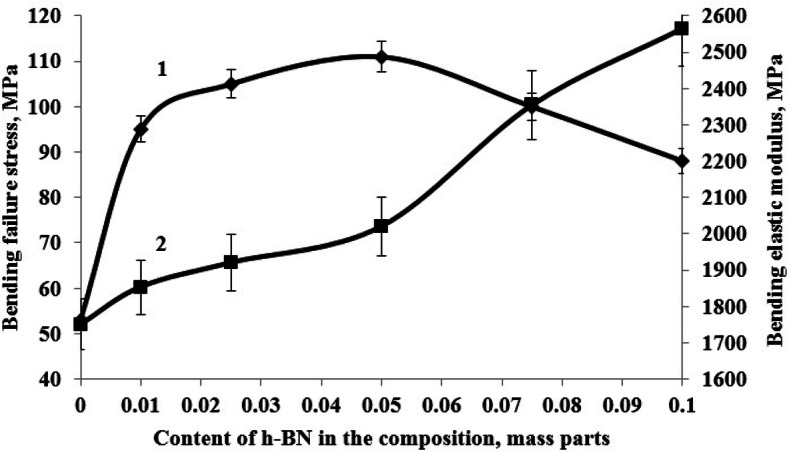
Dependence of bending failure stress (1) and bending elastic modulus (2) of the epoxy composite onh-BN content in the composition.

**Figure 4:**
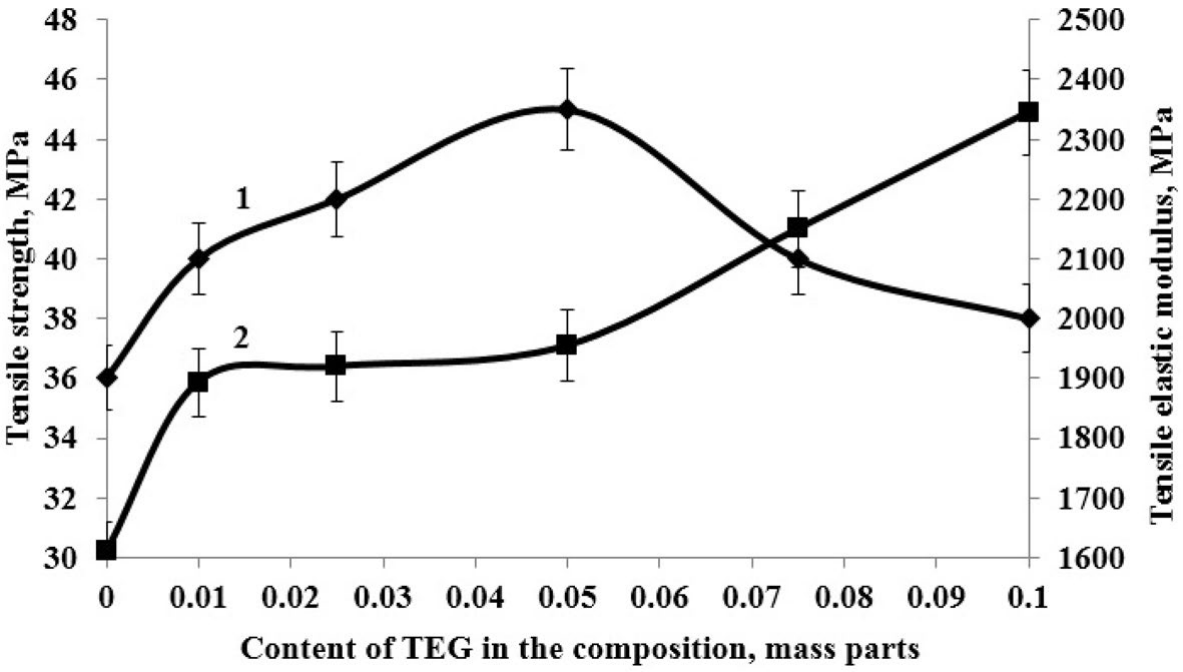
Dependence of tensile strength (1) and tensile elastic modulus (2) of epoxy composite on h-BN content in the composition.

**Figure 5:**
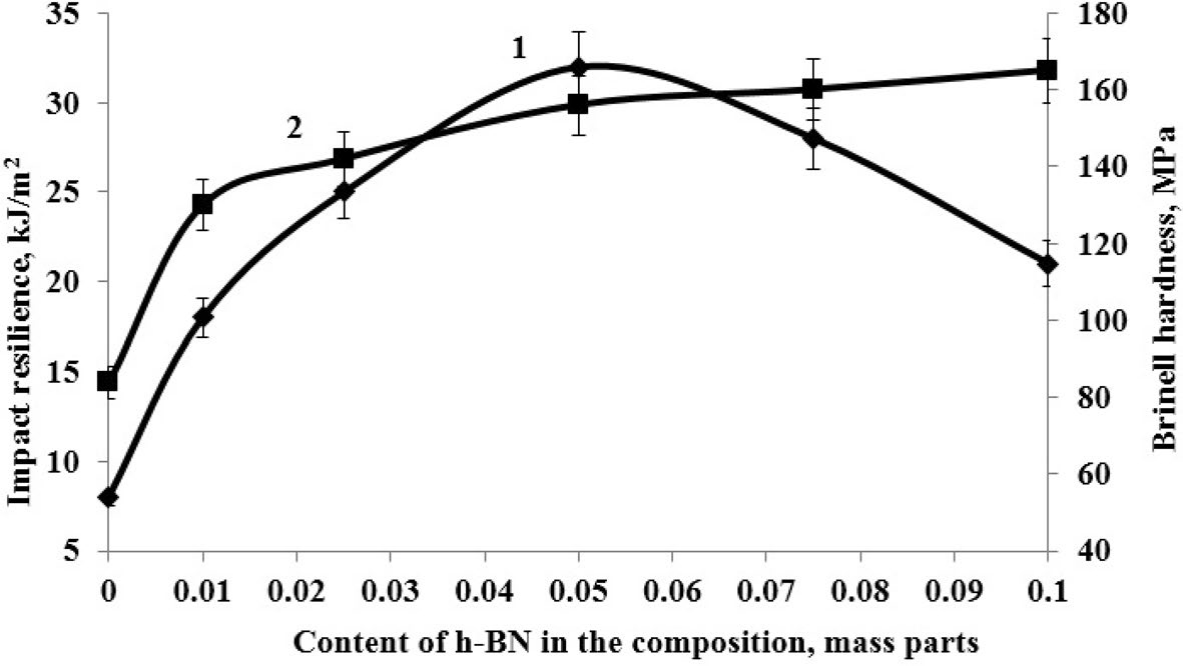
Dependence of impact resilience (1) and Brinell hardness (2) of the epoxy composite on h-BN content in the composition.

The conducted research has shown that the most rational content of h-BN as a structuring additive, providing maximum values of physical and mechanical properties is 0.05 parts by mass, Figs. 3, 4 and 5, at the same time the bending failure stress increases by 109% and the bending elastic modulus increases by 15%, the tensile strength increases by 25% and the tensile elastic modulus increases by 21%, impact resilience increases by 300%, the Brinell hardness increases by 85%.

To ensure the chemical interaction of the mineral filler with the polymer matrix and to increase physicomechanical characteristics of epoxy composites based on them, the h-BN surface was treated with the APTES sizing additive.

To impart hydrophilicity to the surface of h-BN and to make possibie its sizing, h-BN was annealed at 1,100 °C for 1 h. In this case, the main goal of pretreatment of boron nitride powder is to create a sufficient amount of hydroxogroups on its surface, which, when further treated with a silane modifier, will lead to the deposition and fixing of the modifier molecule on the h-BN surface.

**Figure 6:**
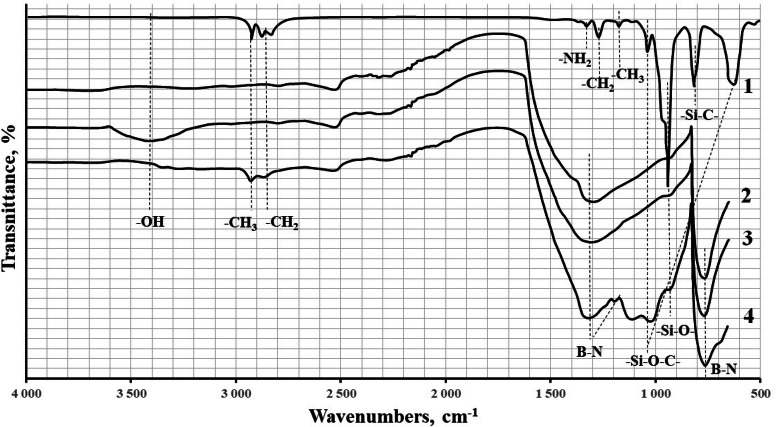
FT-IRspectroscopy: 1—APTES; 2—pristine h-BN; 3—h-BN annealed at 1,100 °C; 4—h-BN after APTES treatment.

**Figure 7:**
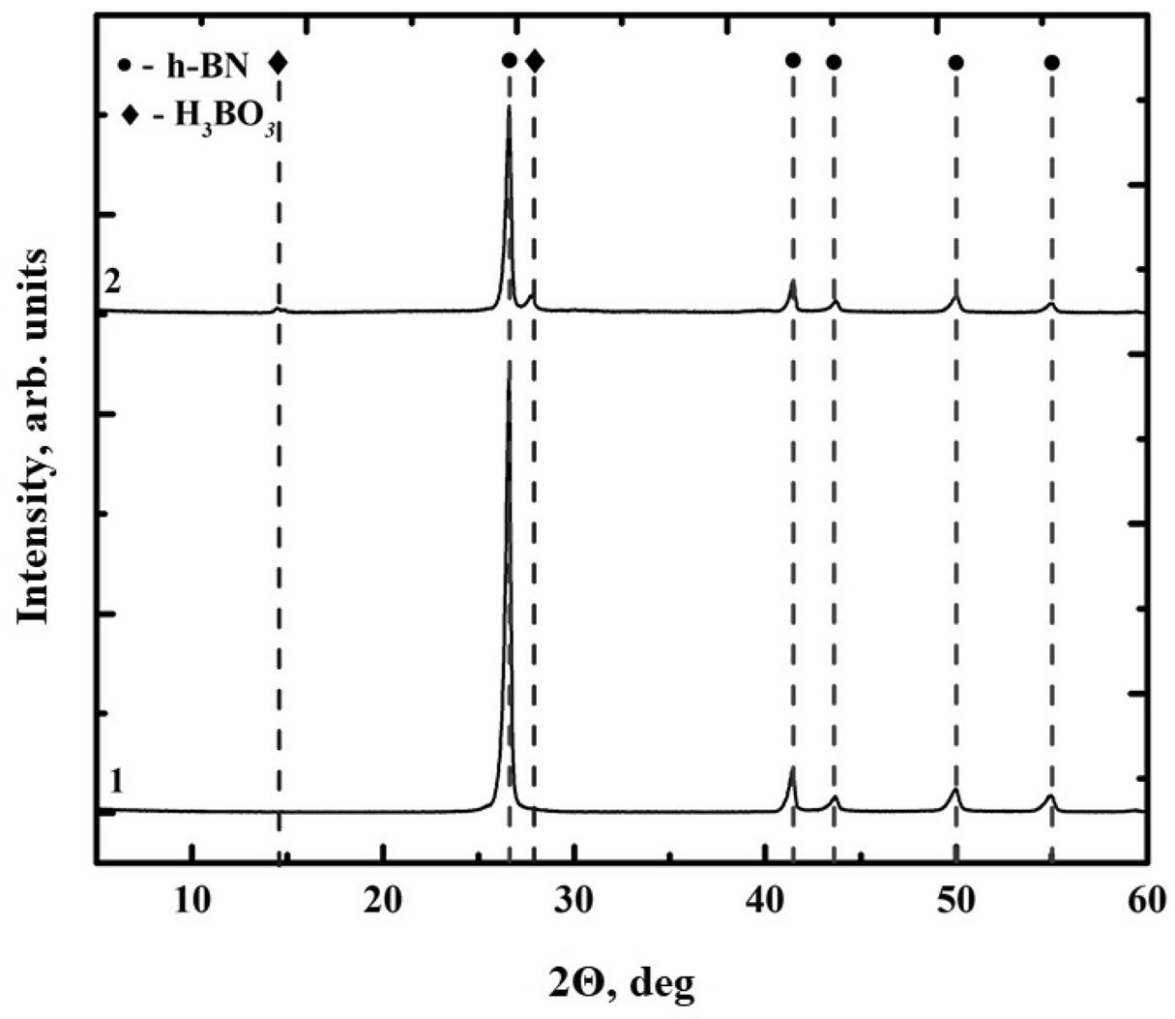
Data of X-ray phase analysis: 1—initial h-BN; 2—h-BN annealed at 1,100 °C.

Hydroxyl surface group formation was verified using FT-IR spectroscopy, Fig. 6, and X-ray phase analysis, Fig. 7, methods. On the IR spectrum of the h-BN sample annealed at 1,100 °C, a peak appears in the 3,400 cm^−1^ range, which is characteristic of the OH group. Besides, a broad line with a maximum of about 1,300–1,350 cm^−1^ also increases, which also indicates the formation of bonds B-OH.

The results of the X-ray phase analysis, Fig. 7, confirm the formation of a stable layer of boron hydroxide on the surface of the material. The diffraction pattern of the sample annealed at 1,100 °C clearly shows the presence of the most intense line of the B(OH)_3_ phase, as well as some other lines of lower intensity characteristic of this phase^20^.

Thus, on the base of all the data obtained, it can be concluded that annealing the initial h-BN powder at 1,100 °C for 1 h leads to the formation of a hydroxide phase, presumably on the fine faces of h-BN flakes, in the amount sufficient for the subsequent treatment. This is a simple and rather effective method of hydrophilization of the surface of hexagonal boron nitride.

The formation of strong bonds between h-BN and APTES was proved by IR Fourier spectroscopy, Fig. 6. As can be seen from the spectrum of the sample after modification, the line of hydroxogroups (3,400 cm^–1^), which is involved in the formation of the APTES layer, disappears almost completely. After APTES treatment, the peak separation in the range of plane stretching vibrations of the B–N bond (1,400 cm^–1^) can also be noted in the spectrum of the sample. The peak separation in this area may indicate an increase in the ordering of the h-BN structure as a result of the material surface treatment. One can also see a broad maximum of about 1,100 cm^–1^, which indicates the presence of Si–O bonds in the sample.

**Figure 8:**
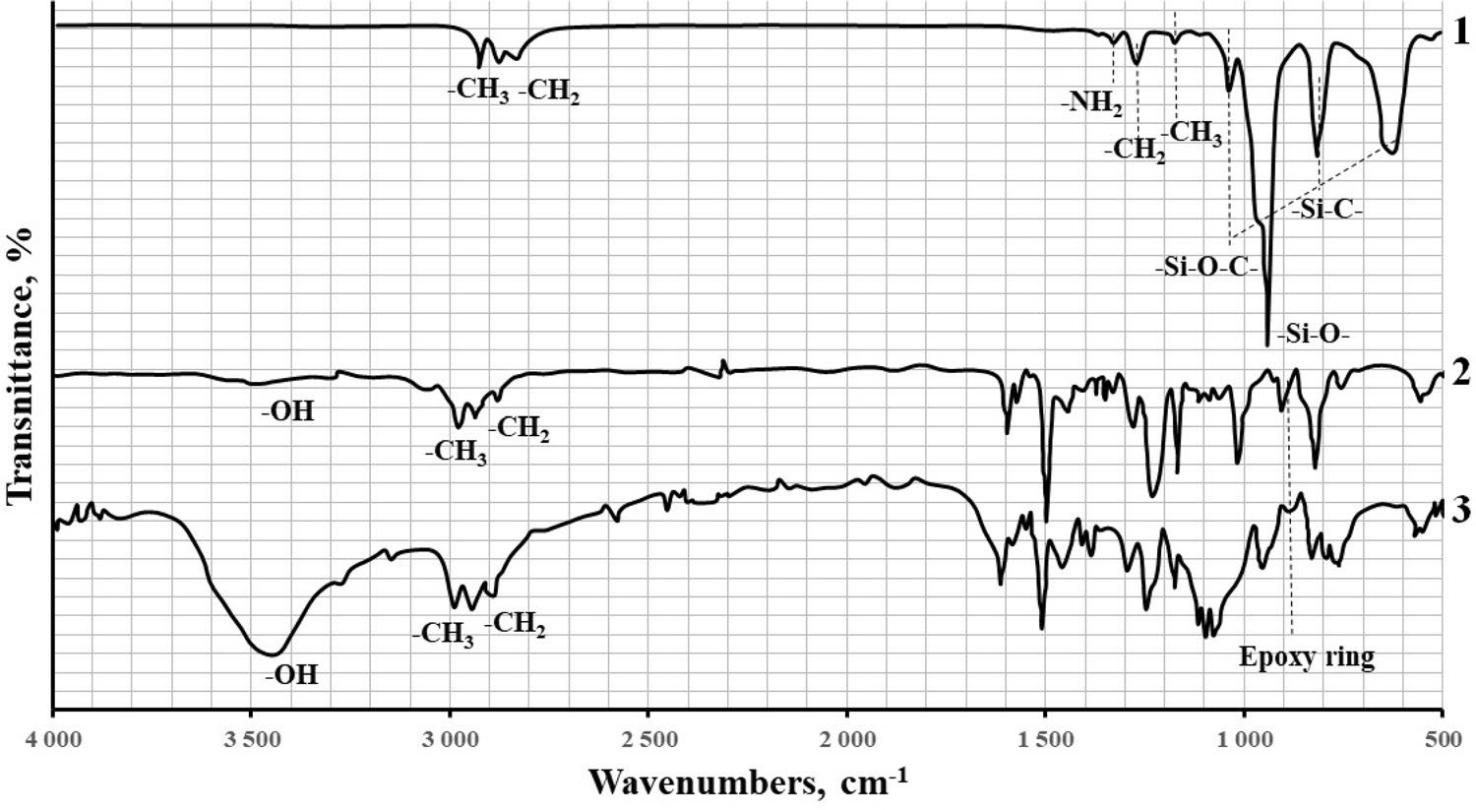
FT-IRspectroscopy: 1—APTES; 2—ED-20; 3—ED-20 + APTES.

The chemical interaction of the APTES functional groupsand the epoxy oligomer was also proved by IR Fourier spectroscopy, Fig. 8.

APTES contains an amine group that can interact with the epoxy ring of the resin when added into the composite. After mixing APTES and ED-20, in the absence of PEPA, a significant decrease in the intensity of the absorption peak corresponding to the epoxy ring (910 cm^−1^) is observed, Fig. 8. This confirms the presence of theinteraction between the APTES amine groups and the ED-20 epoxy groups.

Indirect evidence of chemical interaction is a significant increase in the physic-mechanical properties of epoxy composites, which is possible only when organizing chemical interactions at the polymer matrix/filler interface.

**Figure 9:**
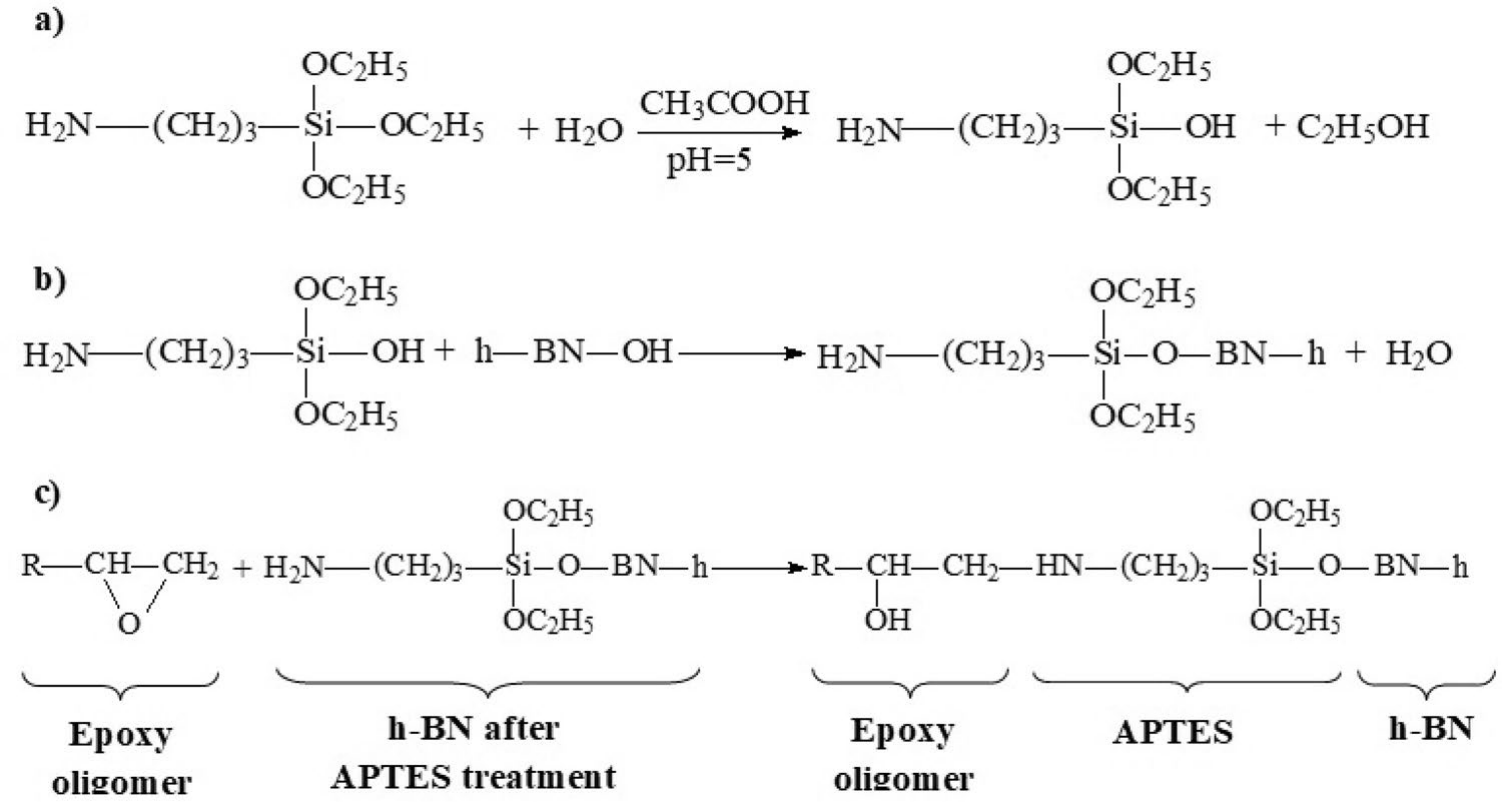
Chemistry of the interaction of epoxy oligomer ED-20, APTES and h-BN: (**a**) preparation of APTES solution in H_2_O; (**b**) chemical interaction of APTES and h-BN; (**c**) chemical interaction of h-BN after APTES treatment with epoxy oligomer ED-20.

Based on this, it can be assumed that the reactions shown in Fig. 9 take place in the system.

APTES treatment ensured the delamination and breaking of h-BN particles with a large lateral size. The fractional composition of h-BN particles modified by APTES is characterized by a unimodal particle distribution and is represented by particles from 0.1 to 25 μm, with a predominance of particles with sizes of 5–6 μm, Fig. 2b.

**Figure 10:**
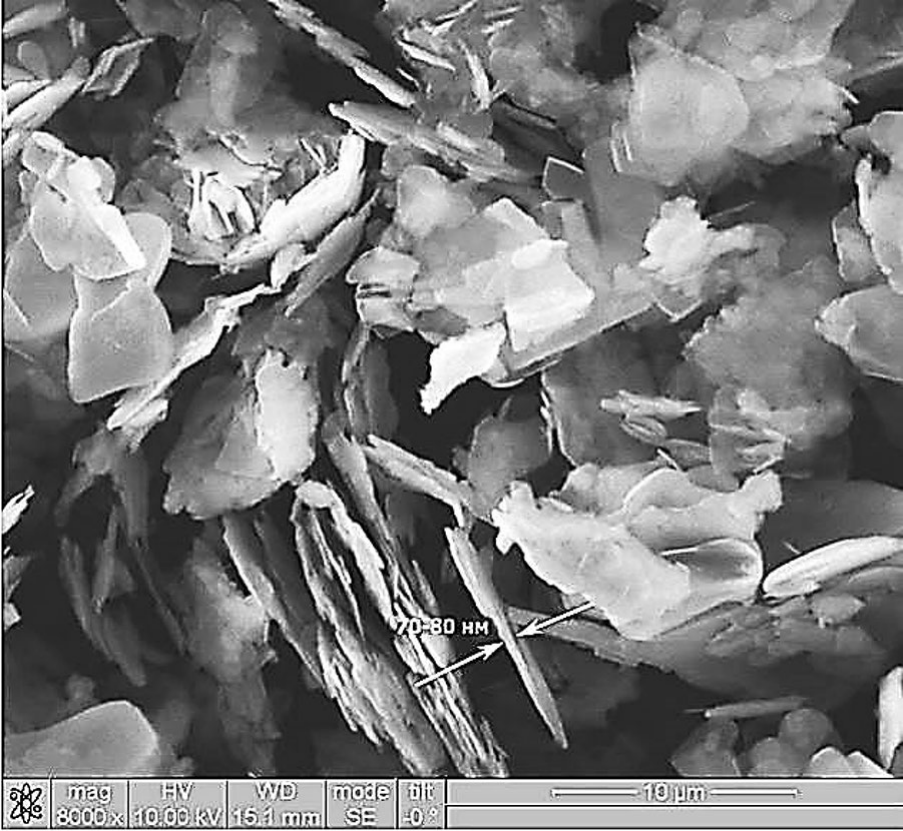
SEM of h-BN particles after APTES treatment.

APTES treatment is accompanied by its intercalation into h-BN layered structures, resulting in the exfoliation of layered structures, which is confirmed by scanning electron microscopy, Fig. 10. h-BN particlesafter APTES treatment are small particles of scaly shape with irregular-shaped edges, 70–80 nm thick.

**Figure 11:**
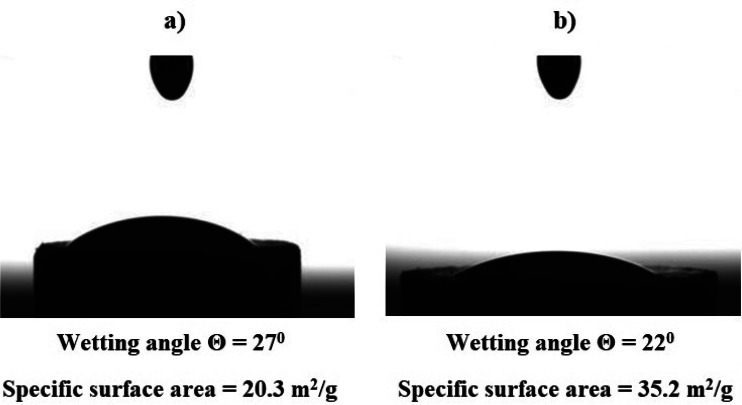
Wetting angle and specific surface area of h-BN particles: (**a**) pristine h-BN; (**b**) after APTES treatment.

After APTES treatment h-BN particles are better wetted with the epoxy composition (ED-20 + TCEP), which is evident in a decrease in the wetting angle from 27° to 22°, their specific surface area increasing from 20.3 to 35.2 m^2^/g, Fig. 11.

**Figure 12:**
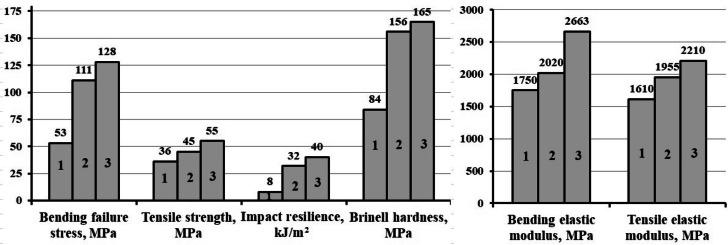
Physico-mechanical characteristics of epoxy composites: 1—100ED-20 + 40TCEP + 15PEPA; 2—100ED-20 + 40TCEP + 0.05 h-BN + 15PEPA; 3—100ED-20 + 40TCEP + 0.05 h-BN_(APTES)_ + 15PEPA.

The organization of the chemical interaction at the h-BN/polymer matrix interface, by treating the h-BN APTES surface, results in an increase in the physicomechanical properties of epoxy composites: bending stress increases by 142% and bending modulus increases by 52%, strength increases by 53% and thetensile elastic modulus increases by 37%, impact strength increases by 400% and Brinell hardness increases by 96%, in comparison with plasticized epoxy composite that does not contain h-BN, Fig. 12.

When assessing the effect of the modifying additive on network polymers, it is necessary to take into account that the curing process takes place in the presence of a developed surface of the solid material (h-BN), which can influence the kinetic characteristics of the polymerization reaction during curing, as well as the formation of the material phase structure. The role of the adsorption interaction of the components of the oligomeric composition with the solid surface of the h-BN is also great^24^.

**Figure 13:**
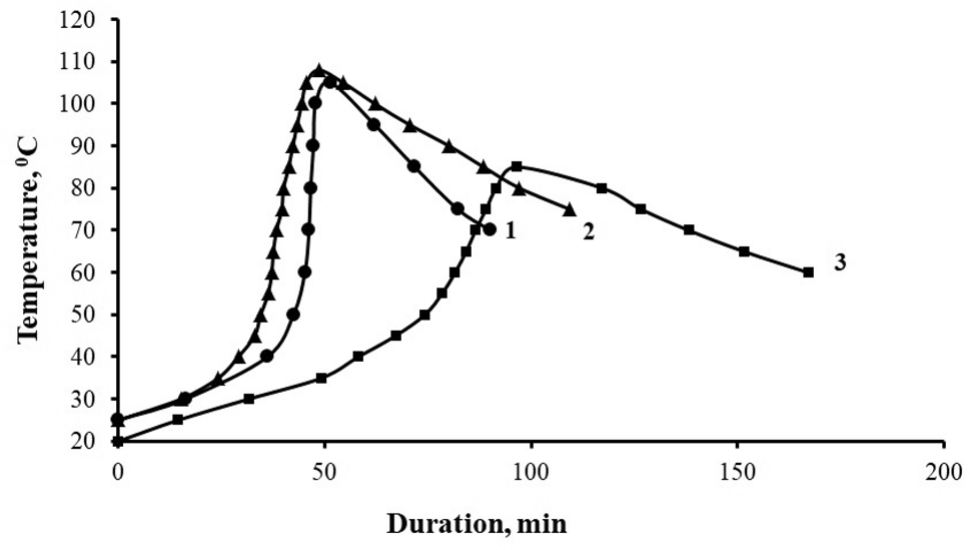
Kinetic curves of compositions curing process, parts by mass: 1—100ED-20 + 40TCEP + 15PEPA; 2—100ED-20 + 40TCEP + 0.05 h-BN + 15PEPA; 3—100ED-20 + 40TCEP + 0.05 h-BN_(APTES)_ + 15PEPA.

The study of the curing kinetics of epoxy compositions, Fig. 13, containing pristine h-BN and h-BN after APTES treatment, show that they have a different effect on the structure formation processes of the epoxy composite, the addition of pristine h-BN reduces the duration of gelation from 45 to 36 min and the duration of curing from 53 to 48 min, while the maximum curing temperature remains practically unchanged, and the addition of h-BN after APTES treatment provides an increase in the duration of gelation from 45 to 74 min and curing from 53 to 96 min, the maximum curing temperature decreasing from 105 to 85 °C, Table 1, which is associated with the formation of thicker boundary layers due to the chemical interaction of APTES functional groups and an epoxy oligomer, which differ in their properties and structure from the bulk of the polymer, leading to a change in the ratio between the components of the binder at the filler surface^21,23^.

**Table 1:** Values of the curing process of epoxy composites.

Composition, parts by mass, cured by 15 parts by mass of PEPA	τ_гeл_, min	τ_oтв_, min	T_max_, °C
100ED-20 + 40TCEP	45	53	105
100ED-20 + 40TCEP + 0.05 h-BN	36	48	108
100ED-20 + 40TCEP + 0.05 h-BN_(APTES)_	74	96	85

τ_gel_ is duration of gelation process, τ_cur_ is the duration of curing, T_max_ is maximum temperature of the self-heating of the sample during curing.

Using the method of thermogravimetric analysis, it was stated that the addition of pristine h-BN and h-BN after APTES treatment does not reduce the heat resistance of epoxy composites based on them, while the Vicat heat resistance increases from 100 to 114–170 °C, Table 2.

**Table 2:** Physico-chemical properties of epoxy composites.

The composition, parts by weight, cured with 15 parts by weight of PEPA	T_i_, °C	T_f_, °C	The yield of carbonized structures at T_f_, %mass	T_v_, °C
100ED-20 + 40TCEP	180	360	55 (360 °C)	100
100ED-20 + 40TCEP + 0.05 h-BN	185	370	54 (370 °C)	114
100ED-20 + 40TCEP + 0.1 h-BN	185	375	55 (375 °C)	122
100ED-20 + 40TCEP + 1.0 h-BN	186	377	55 (377 °C)	160
100ED-20 + 40TCEP + 0.05 h-BN_(APTES)_	186	365	55 (365 °C)	125
100ED-20 + 40TCEP + 0.1 h-BN_(APTES)_	186	370	54 (370 °C)	152
100ED-20 + 40TCEP + 1.0 h-BN_(APTES)_	190	370	55 (370 °C)	170

T_i_, T_f_—initial and final temperature of the main stage of thermolysis; T_v_—Vicat heat resistance.

An important characteristic of materials used in electronic equipment is thermal conductivity. Unmodified epoxy composites have a low thermal conductivity (0.1 W/m K), therefore, when locally heated, they work as thermal insulation, which can lead to overheating and thermal decomposition of the composite^18,25^.

The addition of even small additives of h-BN into the epoxy composition increases the thermal conductivity coefficient by 52–217%, while a decrease in thermal resistance is noted, Table 3.

**Table 3:** The effect of h-BN on the thermal conductivity of epoxy compositions.

The composition, parts by weight, cured with 15 parts by weight of PEPA	The coefficient of thermal conductivity, W/m K	Thermal resistance, m^2^ K/W
100ED-20 + 40TCEP	0.105 ± 0.0058	0.086 ± 0.0043
100ED-20 + 40 TCEP + 0.05 h-BN	0.160 ± 0.0064	0.063 ± 0.0032
100ED-20 + 40 TCEP + 0.1 h-BN	0.186 ± 0.0075	0.042 ± 0.0021
100ED-20 + 40 TCEP + 1.0 h-BN	0.224 ± 0.0090	0.038 ± 0.0015
100ED-20 + 40 TCEP + 0.05 h-BN_(APTES)_	0.165 ± 0.0065	0.060 ± 0.0030
100ED-20 + 40 TCEP + 0.1 h-BN_(APTES)_	0.190 ± 0.0077	0.040 ± 0.0020
100ED-20 + 40 TCEP + 1.0 h-BN_(APTES)_	0.228 ± 0.0091	0.036 ± 0.0014

## Conclusion

As a result of the research, the possibility of the directional regulation of operational properties of epoxy composites using small h-BN additives which provide the creation of epoxy composites with high performance properties has been proved. The effectiveness of the surface modification of h-BN by γ-aminopropyltriethoxysilane and the formation of strong chemical bonds at the polymer matrix/filler interface have been proved, which ensured an increase in the physico-mechanical characteristics of epoxy composites.

It has been confirmed that h-BN particles after APTES treatment are better wetted by the epoxy composition (ED-20 + TCEP).

The study of the curing kinetics of epoxy compositions containing pristine h-BN and h-BN after APTES treatment show that they have a different effect on the structure formation of the epoxy composite.

The introduction of even small h-BN additives into the epoxy composition increases the thermal conductivity coefficient by 52–217%, while a decrease in thermal resistance is noted.

Thus, the developed materials can be used for sealing electronic products, for the impregnation and filling of units in aircraft, ship and automotive industries.
